# Institutional psychotherapy: a conceptual review and clinical toolbox

**DOI:** 10.3389/fpsyt.2026.1857183

**Published:** 2026-07-08

**Authors:** Laurie d’Abbadie de Nodrest, Manon Piette, Habib Bardi, Tudi Gozé

**Affiliations:** 1 Unité de Recherche Culture, Éthique, Religion et Société, Département de Psychologie, Institut Catholique de Toulouse, Toulouse, France; 2 Centre d’Études et de Recherches en Psychopathologie et Psychologie de la Santé - EA 7411, Université de Toulouse, Toulouse, France; 3 Unité de recherche Sciences, Philosophie, Humanités, Université Bordeaux Montaigne, Bordeaux, France; 4 Association pour la Santé Mentale du XIIIème arrondissement (ASM 13), Paris, France; 5 Laboratoire de Recherche sur l’Idéalisme et la Philosophie Allemande Moderne, Université de Montréal, Montréal, QC, Canada; 6 Communauté Thérapeutique La Chrysalide, Montréal, QC, Canada; 7 Pôle de Psychiatrie, Centre Hospitalier Universitaire de Toulouse, Toulouse, France; 8 Équipe de Recherche sur les Rationalités Philosophiques et les Savoirs - EA3051, Université de Toulouse, Toulouse, France

**Keywords:** institutional care, institutional psychotherapy, Marxism, phenomenology, psychoanalysis, psychoanalytic technique, psychosis, transference

## Abstract

This conceptual review introduces Institutional Psychotherapy, a French movement that emerged around World War II as a clinical, political, and theoretical effort to prevent psychiatric institutions from becoming alienating asylums. It extends psychoanalytic practice into a collective, transference-based therapeutic milieu grounded in everyday life. Drawing on psychoanalysis, libertarian Marxism, and phenomenology, we present it as a flexible conceptual and critical toolbox rather than a fixed corpus. We identify two key distinctions and six “logical operators” that structure its originality and discuss its relevance for contemporary psychiatric practice and its tensions with current institutional trends.

## Introduction

Across many contexts, the place of psychoanalysis within institutional psychiatric care has declined (1, 2). At the same time, psychiatry—particularly in the public sector—has been shaped by significant budgetary constraints, leading to increasing emphasis on efficiency and outcome-based evaluations of care, which have tended to marginalize psychoanalytic and other humanistic approaches to the treatment of psychosis. This broader context has contributed to a relative erosion of debates concerning the conditions of psychotherapeutic care for individuals with severe and disabling mental disorders, particularly psychotic conditions.

It is within this situation that the present article seeks to introduce to an English-speaking readership the conceptual foundations and clinical operators of Institutional Psychotherapy (*Psychothérapie institutionnelle*). This movement—at once clinical, political, and theoretical (3, 4)—emerged in the context of the Second World War as one of the most influential disalienation movements, and subsequently developed in a rhizomatic manner across a range of care settings in French-speaking countries. Elaborated in the context of resistance against fascism, and shaped from an awareness of the concentrationary dimensions of psychiatric institutions, the movement aims to systematically identify the totalizing and totalitarian tendencies inherent in collective organizations and psychiatric theorizations. Contrary to the ordinary understanding of the term “institutional”, Institutional Psychotherapy (IP) does not designate a fixed organization model but rather a critical approach to the processes that lead psychiatric institutions to turn into asylum-like forms of organization.

As a method, it generally seeks to prevent *established* assumptions, norms, and codes from sedimenting into mechanical, taken-for-granted rules. In doing so, it attempts to avert the worsening of *psychopathological alienation* arising from psychiatric conditions, by considering the impact of *social alienation*, which is rooted in a therapeutic milieu, itself inscribed in a socio-economic context (5). Conceived as a “political act”, the revolutionary impetus of IP must be constantly renewed; otherwise, routine, habits, and power relationships stagnate and sediment, turning into unquestioned rituals (6). The political dimension of IP cannot be separated from a clinical concern: namely, the conditions under which psychosis can be approached, sustained, and transformed within a collective setting. In this respect, IP advances a decisive hypothesis: the treatment of psychosis cannot be dissociated from a psychoanalysis of the institution itself.

IP thus offers a compelling conceptual history and a set of clinical apparatus and tools for thinking about the psychiatric treatment of psychosis today, making it of particular relevance to contemporary psychoanalysis for several reasons. Contemporary psychoanalysis increasingly recognizes that psychic processes are inseparable from their broader environmental factors. This is particularly evident in psychosis, where clinical experience involves complex interactions between subjects, milieus and political and biological dimensions, rather than intrapsychic mechanisms alone. In this respect, IP proposes a highly original approach to the clinical treatment of psychosis, one that engages with the relational and material milieu while attending to the micro-psychic and experiential movements of everyday life. Second, it represents a form of psychoanalytic practice that was not imported into psychiatry from the outside, but rather developed from within psychiatric institutions themselves. IP extends the core psychoanalytic concerns—such as transference, countertransference, and the conditions of subjectivation—beyond the dyadic setting, toward the collective and organizational dimensions of care, while embedding psychoanalytic ethics within institutional practices. Finally, as a critical intellectual movement, it is structured by a markedly interdisciplinary orientation. At the theoretical intersection of psychoanalysis, phenomenology, and Marxism, IP has demonstrated a striking degree of conceptual inventiveness. Although the epistemological coherence of this approach may at times appear uncertain, its hybrid formations remain consistently guided by a radical clinical ethics that we consider essential to uphold within contemporary psychiatry.

Since conducting a conceptual review of these heterogeneous and interwoven intellectual and clinical histories is not an easy historiographical task, some methodological consideration must be acknowledged. Unlike many psychiatric or psychotherapeutic approaches, IP has never constituted a unified theoretical school, nor has it been defined through a consensual operational framework. Since its emergence it has developed through a plurality of local experiences, theoretical appropriations, and clinical experiments, often resisting attempts at doctrinal stabilization. As Tosquelles himself repeatedly emphasized, Institutional Psychotherapy should not be understood as a fixed method or a reproducible model applicable across contexts. Although Robcis (7) has recently provided an important historical account of the disalienation movement in France, there remains a lack of comprehensive synthesis that brings together its key therapeutic concepts for an English-speaking readership. For this reason, the present article does not aim to provide an exhaustive review of all texts, practices, or authors that could be associated with IP. Rather, it adopts the perspective of a conceptual review intended to identify the major intellectual influences, conceptual operators, and clinical orientations that have historically structured the movement and continue to inform contemporary practices.

The sources selected were chosen according to their historical influence, their role in the transmission of IP, and their relative consensus across its different currents. Particular attention was given to authors whose work played a central role in shaping and disseminating the movement, including Tosquelles, Oury, Guattari, and Fanon. The corpus considered spans from the 1940s to the present day and consists primarily of French-language sources. It includes scientific publications as well as institutional journals, conference proceedings, clinical writings, philosophical texts, and other documents that are often absent from contemporary bibliographic databases. Because of this heterogeneity and the absence of a consensual definition of IP, any delimitation of the field necessarily involves interpretative choices. We therefore organize our review around the three intellectual traditions that most consistently shaped Institutional Psychotherapy—psychoanalysis, phenomenology, and Marxism—while acknowledging that other historical trajectories and interpretations remain possible.

To this end, we first outline the historical development of the movement and its major theoretical influences. We then examine its conceptual “toolbox,” understood not as a prescriptive model but as a set of operators that support clinical thinking and foster inventive praxis within specific institutional contexts. Finally, we discuss the main critiques that can be addressed to IP today and explore its potential contributions to current debates on psychosis, institutional care, and the ethical and political dimensions of psychoanalytic practice (8).

## Historical roots and interdisciplinary foundations

### Historical context of emergence

Institutional Psychotherapy (IP) emerged in France during the Second World War, at the heart of the psychiatric asylum, and later developed alongside the sectorization of French psychiatry in the 1960s. The term itself was introduced retrospectively by Daumézon and Koechlin (9). Its emergence cannot be disentangled from the context of the political trauma: the experience of resistance to fascism intersected with a growing awareness of the segregative and “concentrationary” unconscious of psychiatric institutions, as well as a broader critique of institutional violence.

The early development of IP is closely associated with the work of Francesc Tosquelles (1912–1994), a Catalan psychiatrist who, after fleeing the Spanish Civil War, arrived at Saint-Alban’s hospital in 1940. Drawing on prior experiences of community psychiatry and wartime psychiatric care, Tosquelles, alongside Paul Balvet (1907–2001) and Lucien Bonnafé (1912–2003), contributed to the emergence of collective practices that reshaped daily life. During the Vichy period, these took concrete form in shared activities—agriculture, artistic initiatives—as well as in the development of cooperatives and therapeutic club: the hospital opened onto its surrounding village, thereby blurring established boundaries. At a time when thousands of psychiatric patients were dying of famine in hospitals across France (10), these arrangements enabled Saint-Alban hospital to resist starvation while challenging authoritarian asylum structures. In this context, the asylum was no longer conceived as a space of confinement but as a « refuge » in both an ethical and political sense. Saint-Alban became an intellectual and artistic center, linked to surrealism, attracting figures such as Georges Canguilhem (1904–1995), Paul Éluard (1895–1952), and Jean Dubuffet (1901–1985), combining clinical, artistic, and philosophical innovation. Many psychiatrists trained there and later contributed to the dissemination of IP in other institutional settings.

Jean Oury (1924–2014) founded La Borde clinic in close collaboration with Félix Guattari (1930–1992). La Borde became an emblematic site where democratic institutional practices—meetings, role rotations, therapeutic clubs, and shared responsibilities—sought to reorganize both care and everyday life. At the same time, Frantz Fanon (1925–1961) (11), who had been trained in phenomenology and psychiatry and completed part of his psychiatric training at Saint-Alban, became Medical Director of Blida-Joinville Hospital in colonial Algeria. Initially, Fanon sought to implement the principles of Institutional Psychotherapy and sociotherapy that he had encountered at Saint-Alban. However, he quickly became aware of their limitations when working with Algerian patients. In the colonial context, alienation could not be reduced to the effects of asylum institutions alone; it was also produced by the broader structures of colonial domination that permeated everyday life and social relations.

These practices were supported by ongoing collective reflection within groups such as the GTPSI^1^ (*Groupe de Travail de Psychothérapie et de Sociothérapie Institutionnelles*), which met between 1960 and 1966 and functioned as a genuine laboratory of ideas. The GTPSI sought to renew the theoretical and clinical elaboration of post-war psychiatric institutions and to collectively identify the logical operators of IP in order to consolidate it as a distinct intellectual and clinical movement. It must be acknowledged that all this work has not led to a unified definition of institutional psychotherapy, which has remained diverse and multifaceted to this day. The GTPSI’s central aim was to legitimize the adaptation and transformation of psychoanalytic concepts according to the specific requirements of institutional psychiatric practice (12).

Recent historical research has highlighted the decisive contributions of women to the development of Institutional Psychotherapy, challenging narratives that have often centered on a small number of male psychiatrists (13–15). Before Tosquelles’ arrival at Saint-Alban, Agnès Masson (1900–1993), one of the first women to head a psychiatric hospital department in France, had already initiated significant reforms by humanizing everyday life within the institution, promoting occupational and artistic activities, and reducing coercive practices such as the use of straitjackets (13, 15). Germaine Balvet-Thiébaut also played a key role in the development of care practices at Saint-Alban, combining clinical innovation with an ecological investment in the local environment that proved particularly valuable during periods of wartime scarcity (13, 16). Another important figure was Hélène Chaigneau (1919–2010), co-founder of the GTPSI alongside Tosquelles and Oury, who contributed substantially to the dissemination of Institutional Psychotherapy within French public psychiatry, the development of extra-hospital services, and therapeutic clubs (17). She consistently argued that Institutional Psychotherapy should not become a ready-made model, but rather remain a space for dialogue between singular and non-transferable clinical experiences, capable of sustaining collective creativity (18). Through her involvement in professional networks and editorial activities, she also played an important role in extending the influence of the movement beyond its traditional circles (19).

IP should not be understood as an isolated experiment, but as part of a wider postwar movement within French psychiatry, marked by intense theoretical and clinical debate and by a shared effort to dismantle the alienating structures of asylum psychiatry and to rethink care. In this respect, it resonates with progressive political movements in psychiatry and anti-psychiatry movements that contributed to expanding debates on institutional reform. Although IP remained distinct from these movements, it engaged in ongoing dialogue around questions of institutional authority and patient liberation (see 6). These developments also resonate with contemporaneous analyses by Erving Goffman and Michel Foucault on coercion and surveillance (20, 21).

Today, although IP has declined alongside transformations in contemporary psychiatry, it persists both in historical sites and in new clinical settings, within psychiatric hospitals, therapeutic clubs, and research networks such as the “*Rencontres de Saint-Alban*” and the “*TRUC* federation”. In more dispersed forms, initiatives inspired by IP continue to sustain its ethical and collective orientations in response to emerging social and clinical challenges, often through conflictual dialogue and articulation with recovery-oriented approaches, and community psychiatry.

### The three main influences of contemporary institutional psychotherapy

Institutional Psychotherapy is a heterogeneous movement that cannot be easily categorized in the history of twentieth-century Western thought; however, we can clearly identify three major influences that have a theoretical and practical impact on the key concepts we will present in Part 2. These three influences are: the psychoanalytic movement; Marxist theory and revolutionary practices; and the phenomenological method stemming from German and French philosophy. These influences are part of the conceptual and critical history that continues to inform contemporary Institutional Psychotherapy.

#### Psychoanalytic influence

The elaboration of Institutional Psychotherapy took place within a specific moment in the history of French psychiatry, marked by an unprecedented rapprochement between psychoanalysis and psychiatry, as exemplified by the Bonneval colloquia (see 22) and the post-war activities of the group *L’Évolution psychiatrique*. As Tosquelles (23) writes, the aim was to “introduce psychoanalytic tools into psychiatric settings” (p.76). This involved rethinking psychiatric practice beyond strictly biological and deficit-based models, as well as beyond the asylum framework.

If psychoanalysis is mobilized within IP, it is not primarily as a theoretical doctrine organizing the hospital or care services. While it offers conceptual resources for approaching psychopathological phenomena—especially psychosis—, psychoanalysis in IP is first and foremost an ethical gesture: to welcome the other in his/her singularity, in the irreducible, opaque, and resistant dimension of his/her subjective experience. Here, psychoanalysis supports less a set of techniques than a clinical stance grounded in a wager: that of the encounter, and of the therapeutic effects that may emerge from transferential and countertransference dynamics. Through these processes, the aim is to reactivate subjectivity and to sustain the conditions for a possible psychic movement.

The clinical and ethical stakes of psychoanalysis—historically developed for the treatment of neurosis—are thus displaced in contact with the specificity of psychosis and with the institutional milieu. For Tosquelles (24), the task is not only to think psychoanalysis within the institution, but a “psychoanalysis of the institution” (p.15). These shifts have generated significant theoretical and practical creativity within psychoanalytic thought, which this article seeks to convey.

This elaboration developed within a markedly heterodox conceptual framework. IP never constituted itself as a unified psychoanalytic theory; rather, it emerged as a clinical praxis drawing on a plurality of theoretical references, mobilized as tools for thinking through specific clinical situations. This heuristic attitude—often marked by doubt and by the critical reworking of concepts—also reflects the transformations of the psychoanalytic landscape throughout the twentieth century. Accordingly, the references mobilized by IP practitioners have varied across periods and across the different settings in which it developed, depending on both the intellectual and clinical debates of the time and, above all, on the exigencies of practice. Schematically, we will distinguish between the early elaborations of the 1940s and 1950s, centered around Tosquelles, and later developments, particularly from the 1960s to the 1980s, marked by Lacan’s influence on Jean Oury’s thought.

##### Early developments

At Saint-Alban, the early elaborations of Tosquelles drew on several psychoanalytic references then in circulation: above all the work of Sigmund Freud, but also that of Melanie Klein and Jacques Lacan’s thesis *On Paranoid Psychosis in its Relationships with the Personality* (25). These references played a central role in the development of the conceptual tools mobilized by IP to approach psychosis and to support one of its fundamental wagers: the existence of transference in psychosis.

Freud’s and Lacan’s elaborations on desire are particularly crucial in this respect. Freud (26) proposed understanding delusion not as a mere pathological manifestation but as an attempt at recovery, while Lacan (25) emphasized the internal coherence of delusion. These perspectives opened the way to recognizing a specific logic inherent to psychotic productions and encouraged attention to the singular dynamics unfolding within them. At the same time, Klein’s (27) work on the schizo-paranoid position and mechanisms of projection onto partial objects provided a framework for thinking through the clinical material shaped by fragmentation and dissociation in psychosis. In this sense, splitting and fear of fragmentation were also addressed at the institutional level, in order to support the binding function of the psychiatric hospital (5).

Taken together, these references converge in articulating the specificity of transferential dynamics in psychosis, described as “multireferential” or “dissociated transference” (3). In contrast to the Freudian neurotic situation, where transference is primarily organized within a dyadic relationship, psychotic transference unfolds in diffuse and non-linear forms. It is distributed across a plurality of supports: it may involve not only persons—within an extended network of caregivers and patients—but also objects, spaces, groups, or even animals. Transferential investments thus circulate in a plural and dispersed manner, often emerging within the most ordinary elements of everyday life (28, 29).

This conception also leads to a reconsideration of the fundamental clinical concept of psychoanalysis, particularly the principle of free association, which allows for the emergence of unconscious material. In Freud (30), the analytic frame invites the patient to speak with minimal censorship, so that intrapsychic conflicts may unfold and transform, and so that each subject may trace a singular trajectory beyond any prior assignment to a fixed role. In the institutional context of psychosis, Oury, Guattari, and Tosquelles (5) preserve this principle by making freedom of movement a concrete (material) condition of both psychotherapy and transference that is *a sine qua non* of institutional analysis (31). This possibility of circulation—initially physical and material—also entails a symbolic circulation (the subject’s associative chains). It thus opens the possibility for desiring and creative trajectories within an institutional milieu willing to remain open to the unknown paths of singular elaboration.

For Tosquelles, the complex interweaving of psychotic investments, desires, fantasies, and identifications can only be worked through by taking into account the hospital’s social milieu (7). Interpretation is thus displaced and becomes inseparable from an analysis of the institutional context in which transferential phenomena unfold. The existence of space-times dedicated to the collective elaboration of these transferential dynamics is therefore essential in order to allow the circulation and gathering of the partial investment. Within this perspective, attention to institutional countertransference plays a central role: as Tosquelles (32) emphasizes, identifying and working through the affective movements of caregivers also makes it possible to retrospectively recognize certain defensive expressions at work within the therapeutic relationship. It is therefore essential to organize regular meetings involving the entire hospital staff in order to bring together these scattered fragments of transferential investment. This is what Oury would later conceptualize as the *transferential constellation* (33).

##### Later developments

Building on the initial framework developed at Saint-Alban Hospital, IP continued its practical and theoretical elaboration by drawing on new psychoanalytic theories, in line with transformations in the psychoanalytic landscape and the questions arising from institutional practice. From the 1960s to the 1980s, this landscape was notably marked by the diffusion of Jacques Lacan’s thought, whose influence—already present in earlier developments—became more firmly established in certain strands of IP. This orientation is particularly evident in the work of Jean Oury, who draws on what Lacan (34) described in his 1959–1960 seminar as an “ethics of singularity,” understood as an ethics of incompleteness.

This ethical position distinguishes biological need, which can be empirically satisfied, from lack as a structural condition of desire. Structured around a fundamental lack, desire is expressed through speech and refers to a void that does not require closure or fulfillment. Each subject’s desire is singularized by the way they relate to this inaccessible void. Recognizing this dimension implies, for the analyst, accepting to stand “at the foot of the wall of the opacity of the other”^2^ (35, 36), that is, to engage with the enigma of the other’s desire.

This non-transparency between self and other underscores the need to attend to the unconscious meaning underlying subjective demands, including what remains unspoken. For caregivers, this entails a twofold task: to support patients in accessing their own desire while simultaneously “decompleting” their own therapeutic stance—resisting the impulse to fill or directly respond to what is lacking. Caregivers are thus engaged in an ongoing reflection on their roles, functions, and desires within the institution.

The engagement with Lacan’s work also reinforced IP’s opposition, particularly from the 1960s to the 1990s, to hermeneutic and historically oriented forms of psychoanalysis. Rather than aiming at full abreaction or exhaustive interpretation, Lacan’s (37) work conceives analytic practice as a working-through of the incompleteness of language and the emergences of the unconscious. What has therapeutic effect is not the deciphering or conscious integration of a hidden meaning, but the dynamic effects of speech and what may continue to be said—or to take shape—around an irreducible remainder.

Within this perspective, psychosis is understood not as a global deficit but as a structural configuration of the relation to language and the symbolic order. Lacan (38) notably describes it in terms of foreclosure: certain structuring signifiers cannot be integrated into the symbolic. Psychosis is thus characterized by a disturbance in symbolic articulation, affecting both the possibility of speech and the very conditions of its production. In this light, and as noted above in relation to the “psychoanalysis of the institution,” the aim is not to apply psychoanalysis within institutions, but to think of institutional structures themselves in terms of their symbolic efficacy (39).

The institution can then be conceived as a symbol-generating operator, capable of supporting processes of differentiation. It becomes a space in which positions are distributed and modes of address made possible, allowing for the circulation of speech and the emergence of spaces and times of symbolic inscription. In this regard, Oury (40) evokes the notion of an “institutional tablature”, borrowed from musical vocabulary: not a fixed structure, but a sufficiently flexible symbolic organization capable of sustaining differences in position and preventing both role confusion and imaginary fusion. The institution thus ceases to be merely a material framework or organization of care, and becomes a structuring space in which differentiated positions can emerge, offering subjects points of anchoring within the collective and the institution of language in a broader sense.

In this sense, the reference to Lacan (see 39, 40) contributes to shifting institutional reflection toward the circulation of positions and roles within the collective. Far from a doctrinal application of “Lacanian” theory, these concepts are mobilized as clinical operators for thinking through the symbolic, imaginative and real conditions of care. The rigorous engagement of IP with Lacan’s thought nevertheless remains deeply non-orthodox and grounded in a refusal of any totalizing theoretical system. This freedom is particularly evident in Oury’s frequent articulation between Lacan’s elaborations on the structure of the subject and the work of Gisela Pankow, despite their distinct theoretical orientations. Pankow’s insights into the dissociation of body image and disruptions of embodiment in schizophrenia proved especially decisive in shaping Oury’s work (see 3). According to Pankow (41), dissociation manifests as an impairment of the boundaries and unity of the lived body. In schizophrenia, such dissociation entails a loss of connection between the body and its own history, temporality, and rhythm. As language—encompassing aspects of bodily experience—becomes impaired, the body also loses its ties to the symbolic order governing social relations. Within this framework, the institution is rethought—across its material, mediating, and symbolic dimensions—as a tool for restructuring the image of the schizophrenic body. This restructuring concerns both form—such as the spatial organization of the body and the dynamic relations between parts and whole—and content, including temporal dimensions and the meanings attributed to each part within the whole (see also 40).

In a different vein, the elaborations of IP are also shaped by the influence of Anglo-American psychoanalysis, particularly through group analytic approaches already present in Tosquelles’ work. The contributions of Michael Balint and Wilfred Bion are especially important in fostering a reflection on collective clinical thinking. In *Experiences in Groups* (42) and in his clinical elaborations on thought processes in psychosis (43), Bion explored the dynamics of transference and countertransference within caregiving teams, proposing conceptual tools that enable groups to develop a psychic apparatus capable of “thinking [the patients’] thoughts” (43, p. 127). Group discussions among caregivers are thus seen as essential for metabolizing the complex transferential processes that emerge in psychotic care, allowing the team to remain open to what cannot be immediately named or resolved (4).

The influence of Donald Winnicott (44, 45) is also evident in the attention given to the role of the environment in psychic experience. This perspective makes it possible to conceive institutional care in terms of a holding function—that is, as a sufficiently supportive environment that renders the world inhabitable for the subject and fosters “playing,” understood as the subject’s creative processes. In this context, Oury (46) introduced the notion of *praticables* to designate institutional spaces or apparatus that can be “found-created” in a singular way by patients. These *practicables* may thus function as supports for the emergence of a transitional space when symbolic structuration is fragile.

#### Marxist and revolutionary praxis

If the early twentieth century witnessed the revolutionary emergence of psychoanalysis, another major theoretical influence that reshaped thought during this period was Marxism. Although psychoanalysis and Marxism have often followed divergent, if not at times contradictory, paths, Institutional Psychotherapy may be understood as one of the sites of their productive encounter. One of the central ideas shaping IP is indeed that the material conditions of social life generate forms of alienation, and that the task is to transform them through praxis, understood in a specifically Marxist sense as a transformative engagement with reality.

Tosquelles’ clinical thought was inseparable from his political trajectory. Tosquelles pursued psychoanalytic training alongside revolutionary activity. As a founding member of the Workers’ Party of Marxist Unification (*POUM*), a libertarian Marxist group active during the Spanish Civil War, he witnessed both fascist and Stalinist repression firsthand. These years of socialist and anarchist experimentation in Catalonia, and his immersion in collective life during the wartime period, shaped his enduring suspicion of bureaucratic and authoritarian structures and led him to develop the idea that meaningful psychiatric transformation must remain in constant movement, lest it turn into rigid, institutionalized protocols. These intense conditions also enabled him to develop revolutionary know-how and libertarian social practices that profoundly shaped both his thought and his medical practice.

This perspective led him to formulate what would become a central concept of IP: the “double alienation,” combining the Marxist notion of social alienation with psychopathological alienation inherent to severe mental illness. For Karl Marx, alienation (*Entfremdung*) refers to the condition in which workers become estranged from the products of their labor, the labor process, their own human potential, and from others. Under capitalist conditions, labor is external and coerced, no longer an expression of one’s essence but a means of survival. As a result, human activity loses its creative and social meaning, leading to a fundamental disconnection from self, others, and the world (See 47). For Tosquelles (31) mental suffering cannot be understood without taking into account the material and social assemblages of life of the patients and the forms of alienation these configurations produce. Institutional care, therefore, must also address these broader conditions. Without such an analysis of the material dimensions shaping the experience of madness, social alienation will inevitably exacerbate psychopathological alienation.

While this perspective may be articulated today in terms of broader social and material assemblages, in Tosquelles’ time these conditions were concretely concentrated within the psychiatric hospital itself. It is therefore within this institutional setting that his critique of social alienation took shape. In an attempt to remain both conceptually grounded and faithful to institutional practice, Tosquelles (48) approached social alienation through the risks of concentrationary organization, hierarchical rigidification, and the human and material shortages of the post-war period. Tosquelles’ (48) insight was that, social alienation affects not only the patients subjected to the totalitizing regime of the asylum but also the caregiver staff. Nurses and doctors may find themselves deprived of their subjectivity, freedom, and ethical responsibility due to routine, procedure, prejudices, hierarchical organization, or fear. Broadly speaking, social alienation occurs in the therapeutic context when individuals mechanically perform their duties, working “as usual”, simply doing “what is to be done”. Social alienation thus becomes a mutual condition, where both patients and caregivers are locked into their roles. IP aims to mitigate this by introducing elements of play and responsibility into the performance of roles within the collective.

The acknowledgment of this dual alienation implies that IP must walk on “two legs” (24): a psychoanalytic leg, to tackle psychopathological alienation, and a Marxist and critical leg to conduct the political analysis of collective functioning. This articulation implies a heterodox psychoanalysis attentive to the material conditions shaping subjectivity, alongside a heterodox Marxism that does not overlook singular experience or the lived subjectivity of actors—dimensions often obscured in more orthodox Marxist frameworks.

​​Addressing double alienation is both a political and a clinical act, involving the collective reappropriation of the material conditions of existence and of decision-making. While the notion of “material conditions” provides a broader framework, in Marx (47) it is inseparable from the organization of economic life—an insight that is crucial for thinking through practices of collective self-management. Tosquelles (49) introduced to this end the practice of the “therapeutic club” (see below “Club Function”) precisely to create a democratic and horizontal space in which the economic determinations of hospital life could be collectively addressed. In the context of wartime scarcity during the Second World War, this dimension was not merely theoretical but vital. Therapeutic clubs, in this regard, can be understood as sites of reappropriation of the conditions of production and exchange. Such reappropriation has direct clinical effects: by modifying the patient’s position within the circuits of exchange, it enables a shift from dependence toward forms of active participation thereby opening the possibility of a renewed relation to others, to activity, and to oneself.

This dual approach is what distinguishes IP within the French landscape of critical and social approaches to psychiatry. IP occupies a distinctive position at a remove from both classical post-freudian psychoanalytic approaches and explicitly communist orientations of social psychiatry (50).

For the more traditional psychoanalytic positions—such as those associated with the *Société Psychanalytique de Paris* (i.e. Serge Lebovici, René Diatkine, or Paul-Claude Racamier)—the care setting must remain largely structured by the traditional analytic frame. They rejected the very idea of an analysis of the collective institution. For these authors, psychoanalysis was to remain the prerogative of the analyst, outside the daily life of the collective and grounded in a dual therapeutic relationship. This rupture became more explicit with the rupture of the Sèvres group (1957–1959), following Oury’s (51) intervention emphasizing the role of psychiatric nurses. For Oury (51), the nurses fully participate in the therapeutic process; they are part of the “transferential constellation” (see below), which implies the necessity of a psychoanalytic orientation in their training.

On the other hand, the French disalienation movement was divided between those open to psychoanalytic and libertarian approaches, and those aligned with the French Communist Party (e.g., Bonnafé, Le Guillant), who adopted the anti-psychoanalytic stance promoted under Stalin (52). This division gave rise to tensions within public psychiatry, which largely embraced sociotherapeutic models focused on the social determinants of mental illness. By contrast, Institutional Psychotherapy emphasized the dual nature of alienation in psychosis.

This dual approach also distinguishes IP from other deinstitutionalization movements in Europe such as English antipsychiatry and Italy’s *Psichiatria Democratica*. In England, key antipsychiatric figures were David Cooper (1931–1986) and Ronald David Laing (1927–1989). Cooper, a Marxist psychiatrist, critiqued the medical model for masking social and political alienation, while Laing (53, 54), drawing on phenomenology, emphasized the formative role of environment and relationships in the self’s development. Together, they pioneered therapeutic communities, notably Kingsley Hall in London. In Italy, *Psichiatria Democratica*, led by Franco Basaglia (1924–1980), combined phenomenology, existentialism, and Gramsci’s “revolutionary reform” with Foucault’s critical approach (55, 56). Their activism led to the 1978 Law 180, dismantling psychiatric hospitals (57). While close in spirit, IP practitioners critiqued these movements, arguing that closing hospitals alone does not abolish the asylum, which endures in social attitudes and the exclusion of the mentally ill. They also warned against ignoring the irreducibility of psychopathological alienation, leading to harmful outcomes like homelessness or incarceration following rapid deinstitutionalization (58).

Beyond the articulation between Marxism and psychoanalysis, the work of other Institutional Psychotherapy practitioners—such as Félix Guattari (1930–1992)—continued to explore this intersection through other pathways, at the crossroads of institutional practice, political engagement, and theoretical innovation. Guattari worked at La Borde alongside Oury, while collaborating with Gilles Deleuze to L’Anti-Œdipe (59) and Mille Plateaux (60) in a radical critique of Freudian psychoanalysis and its normative frameworks. This attests to the open and evolving character of IP: not a fixed reference to Marxism and Freudism, but a series of ongoing reworkings, as exemplified by Guattari’s thought, and, in a different register, by Fanon’s analysis of colonial alienation (61). These political and philosophical currents continue to fuel the ongoing critique—and crisis—of IP’s clinical theory and practice.

#### Phenomenological critical methodology

Institutional Psychotherapy combines psychoanalytic analyses of individual alienation with Marxist critiques of social alienation. However, a full understanding of the specificity of IP’s clinical operators also requires considering the influence of phenomenology on its conceptual apparatus.

Phenomenology entered French psychiatry through Edmund Husserl’s 1929 lectures about *Cartesian Meditations* (62) and Eugene Minkowski’s Lived Time (63). Post-war France saw tensions between psychoanalysis and phenomenology: Karl Jaspers and Eugène Minkowski critiqued Freud’s unconscious, while Lacan, drawing from Martin Heidegger, challenged the notion of a unified psychopathology. Only later, with Maurice Merleau-Ponty’s influence and Husserl’s late work, did phenomenological approaches to the unconscious and the lived body gain relevance in psychiatry (64). From its inception – Tosquelles arrived in France already versed in German phenomenological psychopathology – IP brought together psychoanalysis and phenomenology, despite the strong divisions between these fields in academic settings. It is notably the methodological gesture of the epoché—the suspension and critical interrogation of what appears as natural self-evidence *Selbstverständlichkeit*—that drew the interest of IP practitioners for questioning psychiatric norms and asylum structures (64). In retrospect, one might say that they developed a highly original way of rethinking the epoché, not as a philosophical method but as a radical clinical, ethical and political gesture.

The epoché can be identified, more or less explicitly, in several core gestures of IP. First, in its critique of psychiatric positivism—whether biological or psychoanalytic—and, at the same time, in a decentering of the psychological “subject” in favor of an analysis of modes of being-in-the-world. In contrast to phenomenological psychopathologies that have focused on so-called “deficient forms of human presence” (65), IP has paid particular attention to what enables a re-opening of existential possibilities, in a radically non-normative manner. It has sought to create spaces capable of accommodating the singularity of the experience of madness, without aiming to reduce or eliminate it. This orientation was supported by a heterodox and original form of phenomenology, shaped by influences from anthropological phenomenology and aesthetics. In this regard, particular mention should be made of the collaborations of Jean Oury and Danielle Roulot (1943–2019) with phenomenologists such as Henri Maldiney (1912–2013) and Jacques Schotte (1928–2007), who integrated existential and anthropological phenomenology.

The epoche also unfolds in a second dimension, as a suspension that allows the structures of everydayness to be disclosed, revealing the pre-reflexive condition of the shared world (*Lebenswelt*). Such an orientation explains IP’s sustained attention to atmospheres and to the subtle tonalities that shape lived experience. Maldiney (66) emphasized the aesthetics of experience of lived space and rhythm of affective tonalities drawing on Viktor von Weizsäcker (1886–1957) and Erwin Straus (1891–1975). Through him, IP adopted attention to a “pathic space” (67).

This attention to the pathic dimension of existence is associated with a third gesture: an ethical stance in the encounter of otherness. In this sense the epoché becomes a suspension of anticipatory frameworks that makes possible an openness to alterity as “event”. This is reflected in the conceptualization of the “event,” drawing on both Heidegger’s *Ereignis* (68) and Maldiney’s (66) notion of *l’événement*. It is grounded in an attitude of radical openness and ethical hospitality—drawing on Levinas (69)—as well as in an aesthetic sensibility inspired by Maldiney’s (66) concept of the “open”, both of which are central to IP. The clinical practice of IP thus involves a careful analysis of micro-events unfolding within everyday life, as the very site of the emergence of singularity. It is under these conditions of radical welcoming that the alterity of psychosis can be rendered perceptible and listened to in a non-normative way.

A fourth dimension conceives of the epoché as a critical gesture toward social and political norms. The German phenomenological tradition, though influential, has been critiqued in Anglo-Saxon literature for its reactionary tendencies (70). In post-war France, however, phenomenology took a critical turn with thinkers like Jean Paul Sartre, Simone de Beauvoir, and Merleau-Ponty, who engaged with capitalism, patriarchy, totalitarianism, and colonialism. The second generation of phenomenological psychiatrists— Arthur Tatossian, Wolfgang Blankenburg, Basaglia, and Laing—extended this critique to psychiatric institutions, addressing the marginalization of the mentally ill. A key figure in this critical orientation of IP is Fanon (71–73). In *Black Skin, White Masks* (11), he drew on phenomenology to analyze the lived experience of Blackness in a racist society. His analyses reveal how the body and perception are structured by racialization, and how the supposedly shared world is in fact organized by implicit norms that exclude and distort certain forms of existence. Phenomenology thus becomes, in his work, a critical tool: not only to describe experience, but to expose the normative structures that shape it. This phenomenological analysis sustains Fanon’s critique of colonial psychiatry and his clinical practice. Confronted with the limits of IP in a colonial context and with the racist assumptions of local psychiatry, he developed culturally adapted practices and came to see that psychic de-alienation required political struggle against colonial oppression. His subsequent commitment to the Front de Libération Nationale reflects this shift. While widely recognized as a decolonial thinker, Fanon’s work remained rooted in clinical practice and in the effort to address both social and psychological alienation (7), positioning him as a precursor of contemporary critical phenomenological psychiatry (61, 74). Phenomenology thus remains a critical tool in IP, complementing Marxism’s analysis of power and psychoanalysis’s exploration of subjectivity, forming a third dimension of critique in psychiatric theory and practice.

The three historical influences of IP were, each in their own way, markedly heterodox and situated outside academic circuits: a psychoanalysis oriented toward psychosis, a libertarian and anti-authoritarian Marxism, and a phenomenology decentered from the individual subject. These three influences have merged in complex and context-dependent ways across different IP settings, closely aligned with the needs of therapeutic practice, yet not without posing epistemological tensions. We will now outline some of the key concepts that intersect these fields in order to clarify their interrelations.

## Key concepts and logical operators

IP is not merely an applicable method or a protocol that applies universally, regardless of the socio-historical context. It serves as a conceptual and critical toolbox that aims to facilitate the examination of clinical practices and the analysis of organizational and intersubjective frameworks. It offers what Oury (75, pp. 19-20) calls “conceptual tools” or “logical operators”, which are designed to prevent practical and theoretical concepts from becoming stagnant, ensuring they do not appear ahistorical or decontextualized.

First, we will outline the key distinctions that clarify the relevance of logical operators in the clinical context. IP practitioners insist on making a distinction between institution and establishment, and between status, role and function (76). Second, we will introduce the logical operators that are essential for grasping the theoretical framework of IP and are widely used by its proponents.

### Main conceptual distinctions

#### Establishment and institution

In everyday French language, the terms “*institution”* and “*établissement”* often carry very similar meanings. The authors of the IP have used this semantic proximity to highlight a subtle dialectic. *Établissement* (establishment) represents what is established, including the walls of the hospital, the prevailing laws of the state, internal hospital regulations, and the defined hierarchy among professionals by their administrative rank. It also pertains to our role identities that can be established roles that are experienced as assigned places in the social world: doctors, nurses, or patients. Conversely, the *institution* refers to what always goes beyond the established (see 77). It encompasses the unexpected dynamics in intersubjective encounters that exceed socially defined or prescribed roles (See 78). At its core, the institution is an ethical and collective promise, a commitment to take part in a common world.

A human collective is always constituted in a dialectical movement between what is established and what is instituted. Although it is always-already structured on and by an institutional history that has gradually been sedimented over time into the establishment of a local culture, a nation, or even a civilization. Yet, this established legacy can only be meaningfully inhabited by subjects if it remains open to transformation through singular creative acts, the circulation of ideas, and playful engagements with language and collective practices. The establishment—including the spatiotemporal materiality of the hospital, the political contracts and social roles—provides a necessary foundation, albeit sometimes restrictive, for the institution to emerge and flourish. However, an establishment that rigidly protocols and monitors every human movement, leaving no room for institutional dynamics, risks becoming a reified, uninhabitable space. The “death” or collapse of an institutional collective occurs when it forgets its inherent precariousness and contingency (79). On the other hand, an institution that claims no past and therefore lacks a soil sedimented with habits is equally fictional. No institution can exist without some grounding in the established, at the very least in the facticity of a shared language.

Whereas anti-psychiatry amalgamates the meaning of *institution* and *establishment*, regarding institutions as inherently oppressive–madness representing a form of resistance to this oppression–IP views both institution and establishment as necessary conditions for the construction of subjectivity and social organization. Thus, IP actively counters the process by which institutions become entrenched in established practices and theories. By introducing elements of play, shifting habits, and organizing open possibilities for unforeseen encounters, IP ensures that the creative movement of the institution continually re-emerges from the soil of the establishment. This analytical work must be ongoing; without it, practices that initially seemed novel and transformative could turn into taken-for-granted customs and habits, once again leading the collective to become closed off and mechanical.

#### Status, role, function

The difference between an “institution” and an “establishment” also extends to analyzing the position of each individual (caregiver or cared-for) within the group. The *status* refers specifically to the formal job assignment based on one’s training and hierarchical position within the establishment. The *role* is the position assigned by one member of the collective (e.g., a patient) to another (e.g., a nurse). The role may not align with the hierarchical status. The uniqueness of a person’s role for another is shaped by the conscious or unconscious investment that occurs between the two. The “function” refers to a potential gesture that belongs to no one individually but emerges from the events that take place within the collective. It is characterized by its ability to circulate and be shared within the collective. A function can be embodied by a small group or by a single person, often emerging spontaneously without explicit intent, to produce effects on the collective. In this way, the caregiving function is neither the prerogative of the caregiver nor the patient; it is precisely a function, a potential that is neither owned nor exclusively actualized by any individual or established status (80). Within this dynamic, each person is responsible for maintaining a certain distance from these three forms of institutional investment, so as to avoid excessive identification with status—what Oury (4) describes as “the ultimate form of madness*”*.

### Logical operators

#### Micro-analysis of daily life

One of the key challenges in IP is to engage with the temporal dynamics of daily life (*le quotidien*). Beyond the routine that typically prevails in hospital settings, the organization of daily activities is often mistaken for mere busywork, constrained by rigid schedules and meticulous planning. Such regimentation risks stifling the spontaneity and fluctuations of daily rhythms, potentially injecting a sense of idleness. To counter this stagnation, IP seeks to foster the lively and relational aspects of everydayness.

The apparent self-evidence of everyday life does not diminish its inherent complexity; its ambiance is intangible and elusive. Therefore, the work of attending to the atmosphere must be constantly renewed to minimize what Oury (81) terms “pathoplasty”—the institution’s tendency to generate pathology or iatrogeny. Unquestioned routines, pragmatic and repetitive programs risk obscuring the atmospheric tonalities of everydayness—the tacit “tuning” of singularities, which is often fleeting, subtle, and unspoken.

By remaining both available (82) and attentive, caregivers can precisely perceive the micro-movements of singularization in patients’ daily life, particularly those who struggle to articulate their experiences through language. Even what might initially seem pointless or insignificant can acquire clinical value if the collective engages in a micro-analysis of what unfolds in daily life. Beyond the content of speech, the intonations, rhythm, and musicality of dialogue are equally significant. It is not just what is said that matters, but how it is singularly articulated within the collective atmosphere. In this context, a smile or any spontaneous gesture—as long as it is not prescribed or expected—can open new pathways for desire (3). Daily life, therefore, is never considered as spontaneously given, but as a shifting, malleable and analyzable social fabric. The central challenge of IP is to sustain these daily movements, no matter how small they may seem, as nurturing them allows therapeutic vectors to be traced and processes of recovery to unfold.

#### Welcoming and holding function

While all models of psychiatric care agree on the importance of being welcoming to people in psychological distress, IP theorizes the welcoming function (*fonction d’accueil*) as one of its main therapeutic vectors. Such an approach is rooted in both psychoanalytic ethics and phenomenological methodology. It involves acknowledging the personal experiences of a subject as they inhabit their lifeworld and navigate the therapeutic process in a singular way, rather than trying to conform them to established and external norms. Welcoming the singular implies that we must avoid relying on what we believe to be “good” for the patient. Instead, the institutional setting must be created and opened up to discover the pathways through which a personal transformation can occur. In practice, a “hollow” frame—one that is not predetermined and leaves room for the unforeseen—may provoke anxiety and raise questions like “what can we do?” or “what must we do?”. Yet, it is precisely this suspension of demands that allows the emergence of a singular position or evolution. It is therefore essential to cultivate this empty space, ensuring it remains free from both explicit and implicit injunctions.

“What the hell am I doing here?” (*qu’est-ce que je fous là?)* is the question that Oury and Tosquelles repeatedly asked themselves and their colleagues (39). Such a question entails that being present alongside a psychotic person is not self-evident, and requires continual reflection. Supported by the institution as a whole, the welcoming function can take various adaptable forms: a committee, a meal, a permanent reception group, or even volunteers responsible for this guiding function. Circulating throughout the institution and transcending each individual status, it challenges traditional host/guest and caregiver/cared-for relations, suggesting that each of us must also allow ourselves to be welcomed. For this reason the function must be upheld by the collective and therapeutic clubs. The welcoming function is not always a direct interaction; it can spread laterally and is mediated through the atmosphere itself.

Welcoming, while preserving the possibility for unpredictable encounters, does not imply offering a completely limitless space. To be sustained over time, this caring availability requires a shared framework capable of receiving and holding expressions of negativity, hatred, and destructiveness. Such holding does not require reciprocity: in this way, patients may allow themselves to be carried before they are ready to carry themselves or others. This “welcoming and holding function” of the collective provides a foundation of trust and reliability, helping to restore what Blankenburg (83) calls “natural self-evidence”.

#### Free circulation and the function of wandering

Free circulation helps counteract the spontaneous “insularity” and the tendency for any group within the hospital to become closed off. However, this free circulation is not automatically guaranteed, even in an open ward; it must be cultivated and elaborated through various practical strategies.

The freedom to move from one space to another allows patients, who may be struggling with symbolization, to access and produce meaning. IP advocates for keeping doors open, enabling patients to come and go, or retreat as needed. Free circulation represents more than just physical mobility; it is the movement of meaning and the process of meaning-making. As previously discussed, in psychosis, free circulation functions similarly to free association in neurosis, highlighting the specificity of dissociated transference.

Yet free circulation cannot exist if all spaces are equally open and homogeneous in their relational and atmospheric qualities. Rather, it requires differentiation, in contrast to the interchangeability and homogenization characteristic of the neo-liberal model of free-market (see 84). Instead, there is a need for differentiated spaces that vary in subjective investment and conditions of access. Free circulation is more than vain wandering; it involves structuring an orientation between distinct spaces and times. Meaning emerges precisely because point A differs from point B. Furthermore, the act of moving from one point to another, and the repetition of these movements of presences and absences, allows for the construction of transitionality (transitional phenomena, transitional spaces).

Among the identifiable forms of free circulation, Guattari (85) also refers to the practice of “rotations” (*roulements*), which involves caregivers regularly shifting the roles they occupy within the institution. The aim is for everyone to be capable of holding various functions while also preserving each person’s singularity. This rotation also helps to open up teams to adjacent wards, preventing compartmentalization, and mitigating feelings of persecution and collective paranoia that can arise within closed groups of caregivers–issues that can have immediate repercussions on the cared-for, even though they may not be directly involved. Thus, both caregivers and cared-for can be “moved” by a change in perspective; the slightest subjective shift becomes an opportunity to potentiate meaning and to challenge the rigidification of the structures that govern institutional life.

Guattari and Ginette Michaud contributed to the development of the concept of *transversality*, which describes the degree of openness in relations, desire, and institutional circulation within a group and across different levels of an institution (86, 87). Transversality functions as a third dimension that both complements and challenges hierarchical, vertical structures of authority as well as the passive spontaneity of purely horizontal organization (86). While Guattari primarily developed the concept as a philosophical and institutional operator, Michaud explored its more explicitly clinical and psychoanalytic implications. For Michaud (87), transversality concerns the circulation of unconscious desire, mediation processes, and the creation of links across differentiated institutional positions. Simply being present within a group is not sufficient to account for the unconscious dimensions of collective life; institutional spaces must be actively worked upon and differentiated in ways that generate varying degrees of transversality (*gradients de transversalité*). These multiple forms of circulation constitute a movement that traverses the institution without ever becoming fully transparent or immediately visible.

In this sense, it designates the very condition that allows movements, displacements, and encounters to circulate beyond fixed positions and closed group dynamics. Fostering transversality means engaging in a collective process of (self-)questioning, acknowledging the precariousness that arises when opening to the other. This involves asking questions such as “What good is it?”, “What does it [*ça*, or the unconscious] think of all this around us?”, or the aforementioned “What the hell am I doing here?”. As exemplified by the practice of rotations, this stance allows for a more authentic interaction between caregivers, cared-for, and their environment; because, as Guattari notes, “so long as people remain fixated on themselves, they never see anything *but* themselves” (86, p. 113).

#### Collective, transference grafts and transferential constellation

The institution cannot continue to institutionalize itself without the work of the “collective” (*le collectif*). The caregiver-cared-for collective is to be understood as a community of heterogeneous singularities. The institutional net is based on the possibility of encountering or withdrawing from the social bond; a possibility sketched by the meshing and free circulation of the collective. Therefore, the collective’s challenge is to propose what Oury (88), after Pankow (89) and Maldiney (66), calls “transference grafts” or “grafts of ‘open’” (*greffe d’ouvert*)—which may or may not succeed, using “cuttings” (90) that may or may not grow into an exchange. The word “graft” does not imply the wholeness or completeness of the transferred entity; it seems appropriate to describe IP de-totalizing clinical practice, where the aim is not to recover a lost totality but to create the conditions for a partial investment by the patient.

This work involves constructing the material and symbolic conditions that enable a partial investment from the psychotic subject. Through group organization and the diversification of the milieu—spaces, times, and activities—the collective introduces openness, relationality, and a degree of unpredictability. Traversing and traversed by a “relational polyphony”, the collective is constituted by a heterogeneity of presences and relations—reciprocal or not. Assuming that each member of the collective is offered a common institutional tablature, the resulting melodic lines do not merely overlap but indeed lead to a polyphony (91). Polyphony assumes that differentiated instruments can coexist, synchronize, or even affirm their presence through silence.

However, a mere collective listening to this polyphony is not sufficient to protect a milieu from its spontaneously pathogenic tendencies. The collective can only articulate singularities insofar as it also assumes responsibility for creating space-times that actively work toward disalienation. In this respect, the collective is tasked with analyzing the agents of alienation (e.g., unquestioned habits, sedimented behaviors, biases) and with gathering the elements of everydayness through institutional apparatuses such as meetings, supervisions, or clubs which introduce thirdness into the environment. Without this disruption (or suspension) of meaning, projective, morbid, or traumatic habits are likely to settle, sediment, and gradually become both operative and invisible.

What can be done to analyze the relational and affective fragments of dissociated transference that occur throughout the collective and institution? The concept of “transferential constellation” (33) implies that the patient’s projections or fragmented investment in the caregiver/cared-for collective can be gathered in a figure or a map (always incomplete) by the caregivers in order to be able to analyze it. The purpose of the “precarious constellation groups” is to elaborate the massive identifications of the schizophrenic subject. As with stellar constellations, we often look for the position of neighboring stars in order to identify one of them, and understand its situation. In practical terms, ““constellation groups” consist of clinical meetings centered on a patient’s situation. Each participant, whether caregiver or not, shares their experience of the situation and their relationship with the group. This setup requires that all members of the care team express themselves freely, without the constraints of hierarchy. Once the precariousness of the situation is explicitly acknowledged, it opens the possibility of engaging with transference and counter-transference. This process encourages caregivers to question their own desire to be present and engage in the institution. Here again, the aim is transversality by preventing tendencies toward cliquishness, cronyism, and paternalism—attitudes that characterize subjugated groups and undermine the effort to foster responsibility and accountability.

The challenge of precarious constellation groups is to set the institutional effort in motion by creating an institutionalizing dynamic with and around a patient. Through discourse, such gatherings put clinical elements back into circulation, potentially leading to a caregiver’s renewed clinical involvement and/or a change of position.

#### Scribe function and the poetics of care

Within IP, particular importance is given to what has been termed the “scribe function” (*fonction scribe*): a function of inscription that ensures that what occurs can be received, recorded and shared within the collective (92). This function does not merely preserve events; it actively contributes to the conditions under which singular productions and effects of sense may emerge. By providing a surface of inscription, the institution enables signs to appear, be sustained, and to enter a field of symbolization and articulation.

In clinical settings, many subjective manifestations appear in coded, cryptic and only partially intelligible forms - fragmentary utterances, gestures, acting-out, repetitive acts, silences, as well as atmospheres, affects, intensities. These pre-symbolic opaque signs, that precede explicit articulation, constitute the very material of clinical work within PI. What remains unclear is not treated as a deficit to be resolved, but as a constitutive dimension of the encounter: not outside language, but as non-discursive modes of address requiring patience and sustained attention.

In line with Lacan’s ethics of singularity (34), this entails taking the time to bear and welcome the other’s enigmatic production in their sometimes brutal or incomprehensible form, without rushing to interpret or impose external frameworks. It involves accepting not understanding. The scribe function thus implies a stance of humility: attending to repetitions, fluctuations, and resonances, discerning elements of countertransference rather than seeking to stabilize meaning into coherent narratives or uncovering hidden meanings. This implies a form of clinical vigilance: the capacity to mark that something is happening, even in its most minimal or barely perceptible form (93).

It also implies recording and sharing these signs within the collective —not as a form of surveillance, but as a way of preserving traces, of marking that something has taken place and of maintaining a collective memory of what has occurred. This ethical stance sustains a temporal and material space necessary for something to unfold. It sustains a collective surface of inscription, a surface on which something can be marked and where heterogeneous forms of saying can persist over time and thus preserves the openness of what occurs, allowing signs to remain available to circulation, return and resonance (80). Inscription is thus not a neutral act of recording but a transformative operation. It introduces a minimal mediation by allowing what has occurred to circulate over time, to become shareable, and potentially revisitable, thereby giving rise to effects of sense. Such effects emerge through institutional affective experience and through processes of repetition, displacement, and rhythmic variation.

Caregivers, in this respect, do not hold an epistemic or hermeneutic privilege over meaning; rather, they assume a collective responsibility to sustain the condition of this scribe function ensuring a stable, differentiated and reliable institutional framework—material, temporal and relational— which enables investments, flexibility in identification and supports the emergence of meaningful trajectories.

From this perspective, the therapeutic logic at work within IP may be described as poetic logic. This does not refer to an aesthetic concern: it concerns what sets the singularity of the subject in motion through phrasing, rhythm, punctuation, or imagery. This poetic function operates less through verbal content than through a certain play in the treatment of speech, situations, and gestures, which reopens possibilities for institutional rewriting. Such a function relies on effects of displacement from literal meaning, opening a space in which something other can be heard in what is said. In this sense, it introduces a play within the very way clinical situations are received and interpreted by caregivers, while also supporting processes of metaphorization for the patient within the play of the collective.

In IP, Tosquelles (94) addresses the poetic function as enabling the emergence of affective and unconscious elements. It allows the subject to access a “half-say” of what may have been repressed, or to participate in forms of creative and spontaneous expression. The poetic function of language is thus a structural dimension through which singularity is continually woven in a dynamic, unpredictable and non-totalizing manner. Here the poetic points toward a dimension of *poiesis*—a transformative and generative process through which new forms of experience, language, and worlds may emerge. In this respect, a “poetics of care” designates institutional spaces capable of sustaining such generative processes.

#### Club function

Therapeutic clubs are a significant part of French psychiatric care history, tied to the role of *associations*^3^ in the psychiatric field. These organizations have played a decisive role in the movement towards disalienation, civil rights and access to citizenship for people with mental illnesses. This enabled *associations* to act as a counterbalance to traditional hospital practices, leading to the creation of hospital committees and the development of both intra- and extra-hospital therapeutic clubs.

The therapeutic club (49) is, first and foremost, a collective associative structure that operates in partnership with a healthcare establishment yet remains autonomous in its economic and administrative functions. Managed by “patients”—who are no longer regarded solely as patients but as active members of the association—it is open to anyone wishing to join. Interactions among club members occur outside the hierarchical and segregated relationships typical of hospital environments, centering instead on the organization of daily life. Activities such as managing the café, organizing workshops, producing a newspaper, coordinating trips, and facilitating work opportunities serve as mediators of daily life, all connected through an interactive network. Members of the therapeutic club actively participate in organizing this associative space through meetings and moments of democratic commitment, such as voting in general assemblies and serving on councils. They assume collective responsibility for decision-making, financial management, and resource distribution. By doing so, it enables sociality and transference grafts to operate singularly for each individual.

Beyond the specific structures of therapeutic clubs, which are historically situated, IP invites us to consider the broader concept of a club function (46). This concept remains highly relevant for understanding the operational dynamics within contemporary care establishments. The club function acts as a mechanism that, without necessarily being embodied in the concrete structure of a therapeutic club, permeates various systems across different times and places. It actualizes in diverse ways to subvert the logics of segregation and domination. For instance, in a psychiatric hospital, the club function might manifest through collective self-management centered around activities like producing a newspaper (and handling its sales) or running a café. Such spaces are underpinned by alternative power dynamics that aim to ensure equality between caregivers and cared-for in the organization of work, allowing everyone—regardless of status—to participate in decision-making about the establishment’s future.

The club function cannot be prescribed *a priori* or reduced to a predefined institutional arrangement. Rather, it must be found and created by patients themselves within a given social and historical context. Frantz Fanon’s experience in colonial Algeria provides a particularly illuminating example. When he initially attempted to implement at Blida-Joinville the sociotherapeutic and mediated psychotherapy techniques developed at Saint-Alban, he was confronted with a relative failure: therapeutic workshops such as theatre groups and hospital newspapers attracted little investment from Algerian patients. In the colonial context, this experience led Fanon to undertake a careful study of local forms of sociality and cultural practices. Rather than reproducing institutional devices developed in France, he sought to create therapeutic spaces that resonated with patients’ everyday lives and modes of participation, notably through music, sports, and Moorish cafés. This experience demonstrated that the club function cannot be dissociated from the cultural and political environment in which it emerges, and that institutional practices must be continually reinvented according to the forms of life they seek to support.

### Critical discussion

Over the course of its conceptual history, IP has developed as a critical instance within psychiatry—one that seeks to provide care *from within* and *through* processes of institutionalization, grounded in singular ecological, social, and cultural contexts (8).

In this discussion, we propose a critical examination of IP by highlighting several limitations that have been identified both internally and by more recent disalienation movements and social psychiatry approaches. This critical perspective is also informed by the authors’ own clinical practice in settings aligned with the tradition of IP, as well as by their direct engagement with the contemporary challenges that shape these institutions.

Such an ongoing gesture of self-questioning appears necessary in order to address, on the one hand, the risk of sclerosis within IP, and, on the other hand, the opposite risk of a dilution of its specificity into a broadly integrative approach, in which the ethical, theoretical, and political force of the movement might be lost.

Beyond the singular dynamics at play in historically emblematic sites—and beyond the impasses specific to each collective, which must always be analyzed case by case—we propose to organize the diverse critiques addressed to IP around three main motifs: 1) nostalgic closure (a critique emerging from clinical and institutional experience); 2) a totalizing and hospital-centered orientation (a critique originating in social psychiatry and user/survivor movements); 3) a tendency to deny systemic forms of oppression while claiming horizontality and universality (a critique articulated within feminist and decolonial psychoanalysis).

#### Nostalgic closure

Although Institutional Psychotherapy was originally conceived as a movement of permanent revolution, its history of practices and ideas has at times been marked by a process of idealization and reification. In our view, this tendency results from two closely intertwined phenomena.

First, one observes among some practitioners of IP a reliance on its historical legacy and on achievements that continue to produce therapeutic effects but are no longer subjected to critical examination. This can lead to a mystification of both practices and theory. In clinical settings, certain actors may come to position themselves as guardians of this myth, contributing to a melancholization of collectives: the present no longer functions as a site of creative reappropriation, but rather as a space of fidelity to an idealized past.

Alongside this dynamic, IP may also tend to isolate itself within the contemporary psychiatric landscape by adopting a position of resistance as a besieged bastion. While resistance to contemporary forms of normalization in mental healthcare—such as neoliberal logics, managerial governance, and evidence-based imperatives—is both legitimate and necessary, these heterogeneous forces are sometimes amalgamated into a single adversary perceived as threatening a supposedly “humanistic” form of care. This can foster an attitude of mistrust and the construction of an external world perceived as hostile and homogeneous, against which one must constantly defend oneself.

This “we versus them” logic may extend to theoretical positioning within IP, taking the form of a nostalgic and fantasized retreat into a so-called “golden age” of psychoanalysis. Such an idealized past is often opposed to developments since the 1990s in French psychiatry, particularly the rise of cognitive approaches and psychosocial rehabilitation, whose epistemological projects are sometimes perceived as directly aligning with broader neoliberal agendas. Within this climate of suspicion, psychoanalysis may come to function as an identity marker, despite the fact that the early psychoanalytic elaborations of IP were deeply shaped by dialogue with a plurality of contemporary theoretical currents.

This posture cannot be understood solely as an internal defensive reaction. It also develops within a broader context in which psychiatric theories themselves are subject to political appropriation. Such dynamics tend to produce an ideological binarization of practices—between a supposedly “humanistic” care on the one hand, and a care reduced to normative or individualistic adaptation on the other. This polarization fixes positions and obstructs critical and interdisciplinary elaboration.

When these intertwined dynamics take hold, they contribute to the reification of practices and theoretical frameworks derived from IP—precisely where they would benefit from being reworked in response to new expressions of social suffering. The critical and transformative praxis of IP then risks solidifying into a stabilized corpus, transmitted through affiliation rather than clinical testing. This may lead to the dismissal of dissonant positions or to a lack of articulation between IP’s conceptual tools and other epistemological stances.

In such contexts, openness to contemporary movements in psychiatry—whether user- and survivor-led recovery approaches, cognitive sciences, Open Dialogue, or certain forms of social psychiatry—no longer serves as an occasion for critical dialogue. The result is a form of epistemic isolation, situating IP at the margins of its historical moment and of the social field it nevertheless seeks to transform, along with an impoverishment of the creative conflictuality that once constituted one of its central driving forces.

This dynamic of reification may ultimately affect theoretical tools themselves, including psychoanalytic and phenomenological concepts. These concepts then risk being mobilized as markers of recognition among insiders, participating in mechanisms of symbolic power—through the use of jargon accessible only to initiates or through a sterile manipulation of operational concepts, detached from their transformative or critical potential.

#### A totalizing and hospital-centered orientation

A second line of critique concerns a paradoxical tendency toward chronicization, which may re-enact a separation between the psychiatric world and society at large. In a protective movement—serving as a shelter or “place of refuge”—and in light of material and economic difficulties that often limit the capacity of collectives to engage beyond their walls, IP may run the risk of concentrating and totalizing the entirety of care practices within a single site. Paradoxically, this may contribute to a form of institutional inertia and spatial segregation of patients.

Collectives initially conceived as counter-powers to the asylum system may thus, at times, withdraw into forms of therapeutic communitarianism—risking becoming enclosed micro-societies and disengaging from legitimate societal expectations (administrative, legal, or governmental) in the name of an ethics of resistance.

Far from being new, such critiques have accompanied the history of IP both internally and externally (e.g. in the work of 95, or 96). They acquire renewed relevance in the contemporary context of deinstitutionalization, promoted simultaneously by public authorities and by critical user/survivor movements. When these political and social tensions are not adequately thought through, they may lead to a sterile dichotomy: on the one hand, an institutional clinic equated with the hospital; on the other, an outreach or community psychiatric practice organized as a neoliberal network that contractualizes and individualizes care.

When collectively elaborated, however, these points of impasse can become valuable clinical reference points for reopening the question of institutional experience and of what is created in common within it. Indeed, engagement in institutional life and its methodological critique extends beyond the material boundaries of the hospital establishment. The social field as a whole is an entanglement of institutions. The task, then, is to interrogate the dynamics of the common, so that they may become experientially accessible and mobilizable within and through the social field.

Provided that human resources allow it, participating in the daily life of a collective thus presupposes the possibility of emancipating oneself from the logic of the refuge, by allowing oneself to be shaped by a plurality of social experiences. From a perspective oriented toward linkage and the overcoming of binary coordinates (inside/outside; shelter/community), the logical operators described above can be mobilized wherever suffering, disconnection, or social disaffiliation call for collective support.

#### Denial of systemic oppression

A third recurrent blind spot—despite institutional analyses explicitly aimed at working through power dynamics—concerns the difficulty collectives may encounter in identifying and thinking through intersecting relations of power, including those related to gender, race, and other forms of systemic oppression. No type of relationship—whether between caregivers and cared-for, or within work relations—is exempt from such dynamics, which may re-emerge through unconscious resistance to institutional analysis.

In their most overt forms, power relations may be discerned in the use of language. Within work relationships, psychiatrists or psychologists often hold the narrative of IP’s history and its theoretical concepts; similarly, knowledge-power may become concentrated within caregiver–patient relations. For this reason, the free circulation of speech, while necessary, is not sufficient. Particular attention must be paid to issues of epistemic injustice, so that each person may be welcomed as a subject collectively recognized as a witness to experience and as a bearer of knowledge.

Although the circulation of responsibility is explicitly articulated, therapeutic clubs may also become sites where unequal distributions of decision-making power operate latently. In atmospheres sometimes shaped by apathy or passivity linked to psychopathology and the iatrogenic effect of medication, caregivers may feel compelled to take initiatives more rapidly, accelerating collective rhythms perceived as stagnating. While such initiatives are often motivated by benevolence or protective intentions, they may nevertheless short-circuit associative statutes that had, through democratic voting, assigned positions of responsibility to service users.

Moreover, psychoanalytic, phenomenological, or Marxist discourses may at times be appropriated for narcissistic or paternalistic purposes within institutional collectives. In response to forms of discourse experienced as overly technical or excluding, many caregivers and users turn away from practices informed by these epistemologies and seek clinical alternatives through a global horizontalization of power. These movements aim to reduce the gap between so-called “knowers” and so-called “ignorant” subjects by emphasizing shared human conditions, teamwork, and common vulnerabilities.

Yet the hidden side of such horizontalizing approaches may involve a denial or invisibilization of power effects, intersecting with multiple layers of systemic oppression. How, then, can one disengage from authoritarianism without falling into the “group illusion” (97) of a fully accomplished horizontality—an illusion that may in fact reinforce a dimension of power by concealing it? Put differently, how might a more hybrid and contemporary IP avoid smoothing over differences in position in ways that mask the ongoing effects of power within institutions? It is a matter of recognizing the asymmetry that necessarily exists between care-givers and those receiving care. These differences in position (which are always dynamic) can become clinically productive operators if, and only if, they are analyzed and rendered visible, so as to prevent their reification into alienating forms of oppression.

Addressing these questions requires sustained attention to the conditions under which a collective voice can remain alive, by working through singularities and through minoritized and situated forms of knowledge, in order to open new pathways of exchange—pathways that resist the rapid sedimentation of universalizing discourses into new organs of power.

#### Limitations and future directions

Several limitations of the present review should be acknowledged. First, because IP does not possess a consensual operational definition and has developed through heterogeneous clinical, political, and theoretical trajectories, the boundaries of the corpus remain necessarily open to discussion. The selection of sources reflects an interpretative effort to identify the most influential and widely transmitted references within the movement rather than an exhaustive mapping of all existing contributions. Consequently, some local experiences, institutional archives, regional traditions, and internal controversies have only been partially addressed or remain outside the scope of the present article.

Second, the documentary corpus associated with IP extends far beyond conventional academic publications and includes a wide variety of materials, such as hospital journals, conference proceedings, unpublished texts, biographical documents, and oral transmissions. Many of these sources are difficult to access through contemporary bibliographic databases, limiting the possibility of applying systematic review methodologies.

Future research could further explore neglected historical actors, particularly women practitioners and caregivers, undertake comparative analyses with other deinstitutionalization and community psychiatry movements, and examine the contemporary transformations of IP in relation to recovery-oriented practices, social psychiatry, Open Dialogue, and current developments in phenomenology and psychoanalysis. Such investigations would contribute to extending the critical and inventive spirit that has historically characterized the movement.

## Conclusion

As Tosquelles famously stated, “*Institutional Psychotherapy does not exist*”, underscoring that IP cannot be reduced to a single model, but rather takes the form of multiple practices, each emerging from local histories and the specific clinical needs of particular care settings or therapeutic communities. In this article, we have sought to capture, as faithfully and comprehensively as possible, the core elements and shared foundations of this protean movement.

The aim of this conceptual review was to introduce key notions and reflective tools that may inspire practices beyond the French context. We suggest that IP has brought a renewed perspective to psychoanalysis and to its relationship with the political and philosophical domains. In particular, it has contributed to displace psychoanalysis toward a collective and institutional praxis, while preserving its ethical core: an attention to singularity and to the transformative effect of address and language.

While this original experience remains shaped by its historical and local limits, it may nevertheless resonate with and inform initiatives across diverse international contexts. Rather than applying these concepts mechanically to practice, our discussion encourages readers to adapt each idea based on their singular historical, political, social, and cultural perspectives. The challenge lies in moving beyond the mythical and sometimes “legendary” figures of IP to prevent a nostalgic reverence for its early stages from reinstating any authoritative functions within a continually reinventing revolutionary movement. In this way, individual and collective de-totalization may be reaffirmed as the fundamental commitment of IP.

Attention to the singular ways of living with and beyond psychosis could serve as a complementary and critical tool to recovery-focused approaches. While experiential recovery may only be assessed from a first-person perspective (by the individuals themselves), caregivers can cultivate a collective ethics of care that supports the emergence of a singular transformation. On the other hand, while the recovery movement has been critiqued for being overly focused on the individual—sometimes veering toward individualism—IP offers an alternative. IP encourages us to think of recovery as a collective process, involving the recovery of a shared and generalized caregiving function within the community.

Perhaps one of the most demanding contributions of IP lies precisely in its capacity to hold together what does not easily align: the collective dimension of care and the singular opacity and irreductibility of each subject. Rather than resolving this tension, IP invites us to sustain it as a condition of clinical and institutional work.

## Data Availability

The original contributions presented in the study are included in the article/supplementary material. Further inquiries can be directed to the corresponding author.

## Footnotes

1 Other groups also contributed to the theoretical development and critical reworking of Institutional Psychotherapy, including the Sèvres Group, the CERFI (*Centre d’Études, de Recherches et de Formation Institutionnelles*), founded by Félix Guattari and active between 1967 and 1987, whose activities were closely associated with the journal *Recherches*, and the international network *Alternative à la psychiatrie*, which fostered exchanges between various movements engaged in the critique and transformation of psychiatric institutions.

2 Our translation.

3 French *associations* are similar to nonprofit organizations or voluntary associations in Anglo-Saxon contexts.
